# Diagnostic Utility of Neutrophil-to-Lymphocyte Ratio in Differentiating Benign and Malignant Ovarian Masses: A Systematic Review

**DOI:** 10.3390/cancers18060960

**Published:** 2026-03-16

**Authors:** Patrick Bayu, Patricia Diana Prasetiyo, Jeremiah Hilkiah Wijaya

**Affiliations:** 1 Department of Obstetrics and Gynecology Fertility Endocrinology, Faculty of Medicine, Pelita Harapan University, Tangerang 15811, Indonesia; patrick.bayu@uph.edu; 2 Department of Pathology Anatomy, Faculty of Medicine, Pelita Harapan University, Tangerang 15811, Indonesia; diana.priyatno@lecturer.uph.edu; 3 School of Public Health and Preventive Medicine, Monash University, Melbourne, VIC 3800, Australia

**Keywords:** benign ovarian masses, diagnostic performance, malignant ovarian masses, neutrophil-to-lymphocyte ratio

## Abstract

Background/Objectives: Given its cost-effectiveness and availability, NLR could play a pivotal role in refining the diagnostic process. We aimed to evaluate whether NLR can serve as a useful marker to differentiate between benign and malignant ovarian masses, synthesizing available evidence to provide a comprehensive assessment of its diagnostic value. Methods: A comprehensive search was conducted across the following electronic databases: PubMed, Europe PMC, and SCOPUS on 4 January 2026. For inclusion, studies were required to report on the use of NLR as a biomarker for differentiating ovarian masses, involve human participants with preoperative measurements of these ratios, and provide data on diagnostic accuracy, such as sensitivity, specificity, or receiver operating characteristic (ROC) curves. Both retrospective and prospective observational studies were considered. Results: This systematic review incorporated data from 3675 patients across ten studies. The meta-analysis revealed that malignant masses exhibited a significantly elevated mean NLR (HR −0.80 [95% CI: −1.17 to −0.42]), but found no statistically significant correlation (OR 1.49 [95% CI: 0.67 to 3.30]). NLR had moderate diagnostic performance with an AUC of 0.66 (95% CI: 0.62–0.69). Conclusions: While malignant ovarian masses are associated with a significantly higher mean NLR, the overall diagnostic performance of NLR remains moderate, suggesting limited utility in distinguishing between benign and malignant tumors.

## Simple Summary

Early and accurate separation of benign (non-cancerous) from malignant (cancerous) ovarian masses can improve referral decisions, reduce unnecessary surgery, and speed access to specialist care. Because a complete blood count is inexpensive and widely available, we examined whether the neutrophil-to-lymphocyte ratio (NLR; a measure of inflammation calculated from routine blood tests) can help distinguish these two conditions before surgery. We systematically reviewed the available evidence and combined results from eight studies including 3675 patients. Overall, malignant ovarian masses tended to have higher values of this ratio, but its ability to correctly classify masses was only moderate, suggesting it should not be used alone to guide diagnosis. These findings highlight the need for better risk models that combine blood-based markers with imaging and clinical features, and for future studies to standardize cut-off values and reporting.

## 1. Introduction

Ovarian mass is a common clinical finding, yet distinguishing between benign and malignant lesions remains a critical challenge in gynecological oncology. Early and accurate differentiation is essential for determining the appropriate course of management, from surgical intervention to the potential need for more aggressive treatment. However, current diagnostic methods, such as imaging and serum tumor markers like CA125 and HE4, have limitations in sensitivity and specificity [1,2,3], hence the pressing need for additional, cost-effective biomarkers that can enhance preoperative diagnostic accuracy.

Currently identified ovarian masses in preoperative works, both benign and malignant, require multifaceted approaches, including clinical, imaging, and biomarker studies [4,5]. Transvaginal ultrasonography (TVUS) is the main imaging method because of its availability, and it can describe the morphology of masses (e.g., presence of septations, vascularity and papillary projections, etc.) [6]. CT and MRI can be used in staging or impending cases; in addition, serum markers (CA125, HE4) and MRI (ROMA, RMI) can be used to obtain risk estimates, though it is recognized that the above mentioned approaches have limited accuracy [7].

The Assessment of Different NEoplasias in the adneXa (ADNEX) model proposed by IOTA has become one of the most widely used tools for predicting multiclass risks [8]. ADNEX combines clinical information with certain sonographic criteria (largest lesion diameter, acoustic shadows, and ascites) to estimate probabilities for the presence of benign, borderline, invasive, and metastatic tumors [8]. While the model is an improvement in patient stratification, operator skill, equipment availability and use of adjunct inflammatory indices is still a requirement to provide greater accessibility [8].

The main difficulty in oncologists who deal with gynecological cancers is figuring out the difference between benign and malignant ovarian growths, as the current techniques like imaging, blood serum tumors, CA125 and HE4, show low sensitivity and specificity for varying clinical situations [2,3,9]. In these instances the neutrophil-to-lymphocyte ratio (NLR) could potentially be a useful additional endpoint with the way it captures the interplay between systemic inflammation and anti-tumor specific immunity [10]. Biologically, the NLR tends to be increased in malignancies and is never related to one single molecular mechanism; it is always representative of the collective phenomena of a greater neutrophil concentration and lesser lymphocyte concentration [10]. Ovarian malignancies, when compared to the benign conditions, are far more likely to provoke a systemic inflammatory response and activate processes like NF-κB and JAK/STAT3, due to the action of tumor and stroma cytokines like IL-1β, IL-6, TNF-α, G-CSF, GM-CSF, and IL-8 [11]. These processes are responsible for the stimulation of blood formation and increased neutrophil concentration, all of which contribute to the NLR elevation seen in malignancies.

Malignant tumors can hinder the function of adaptive immunity, which may explain why there are lower lymphocyte counts compared to benign ovarian masses [12]. The immunosuppressive response to tumors can also explain the increase in NLR as it reduces lymphocyte-mediated surveillance of the tumors [12]. Moreover, tumor-associated neutrophils can also increase angiogenesis, remodeling of the extracellular matrix, and the potential for metastasis (through factors like VEGF, MMPs, and ROS) which just further strengthens the link between high NLR and malignancy [13]. The biological justification of the use of NLR in differentiating malignant and benign ovarian masses is due to its ability to reflect the underlying inflammation of tumors and the dysregulation of immunity [13]. However, NLR is neutral and may also be elevated in infections, endometriosis, and other inflammatory diseases; it is a supplemental marker with imaging, tumor markers, and proven risk assessment models, with none of the singular marking standing on its own for diagnostically measuring/comparing NLR to other malignant [13].

These findings highlight the potential utility of NLR as a supplementary tool in the preoperative evaluation of ovarian masses. Given its cost-effectiveness and availability, NLR could play a pivotal role in refining the diagnostic process, particularly when integrated with existing biomarkers. The objective of this systematic review is to evaluate whether NLR can serve as a useful marker to differentiate between benign and malignant ovarian masses, synthesizing available evidence to provide a comprehensive assessment of its diagnostic value.

## 2. Materials and Methods

The systematic review was performed following the guidelines of the Preferred Reporting Items for Systematic Reviews and Meta-Analyses (PRISMAs) [14]. The protocol for this systematic review was registered with PROSPERO under the identifier CRD420261326995. We received no external funding for running this systematic review.

A comprehensive search was conducted across the following electronic databases: PubMed, Europe PMC, and SCOPUS up to 4 January 2026. The search strategy included the following terms and their combinations (Table 1):

- “Neutrophil-to-lymphocyte ratio” OR “NLR”;
- “Ovarian mass” OR “ovarian tumor” OR “ovarian cancer”;
- “Benign” OR “malignant”.

Search filters were applied to include only studies in English. The reference lists of all included studies and related reviews were manually searched to identify additional eligible studies.

For inclusion, studies were required to report on the use of NLR as a biomarker for differentiating ovarian masses, involve human participants with preoperative measurements of these ratios, and provide data on diagnostic accuracy, such as sensitivity, specificity, or receiver operating characteristic (ROC) curves. Both retrospective and prospective observational studies were considered. Studies were excluded if they were case reports, conference abstracts, or editorials, or if they did not report sufficient data on NLR or focused on conditions other than ovarian masses.

Two independent (PB and PD) reviewers screened the titles and abstracts of all identified studies for relevance. Full texts of potentially eligible studies were retrieved and evaluated according to the inclusion and exclusion criteria. Disagreements between the reviewers were resolved through discussion or consultation with a third reviewer.

Data were extracted by two reviewers (PB and PD) independently using a standardized form. The following data were collected:

- Study characteristics: author, year of publication, study design, sample size, and country;
- Patient characteristics: age, menopausal status, tumor type (benign or malignant), and preoperative NLR value;
- Diagnostic performance of NLR: sensitivity, specificity, area under the ROC curve (AUC), and cutoff values used in the studies.

The risk of bias was assessed using the Quality Assessment of Diagnostic Accuracy Studies-2 (QUADAS-2) tool, which evaluates bias across the following four domains: patient selection, index test, reference standard, and flow and timing. Each domain was rated as “low,” “high,” or “unclear” risk of bias [15].

The data analysis strategy involved calculating pooled estimates for both continuous and categorical variables across multiple studies using a random-effects model to account for heterogeneity. For continuous variables, such as the comparison between benign and malignant ovarian masses, the standard mean difference (SMD) was computed with 95% confidence intervals (CIs), and the overall effect size was tested using a Z-test. For categorical outcomes, like the odds of diagnosing malignant ovarian tumors based on the neutrophil-to-lymphocyte ratio (NLR), odds ratios (ORs) were calculated. Heterogeneity among studies was assessed using the I^2^ statistic, with a high I^2^ value indicating substantial variability across studies. Both analyses incorporated weights based on study size and variance to determine the overall effect. *p*-value was considered significant if the value was less than 0.05.

## 3. Results

**Figure 1:**
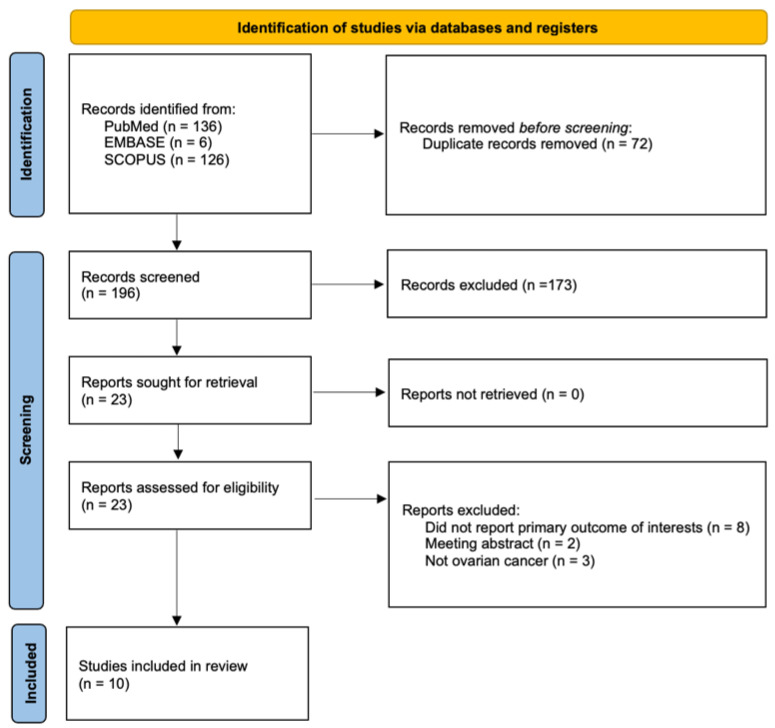
PRISMA flow diagram illustrating the study selection process for this systematic review.

The PRISMA flow diagram (Figure 1) described the study selection process for the systematic review. Initially, 268 records were identified across three databases: PubMed (136), EMBASE (6), and SCOPUS (126). Prior to screening, 72 duplicate records were removed, resulting in 196 records that were screened. Of these, 173 records were excluded based on the eligibility criteria. A total of 23 reports were sought for retrieval, and all were successfully retrieved and assessed for eligibility. However, 13 reports were excluded—eight for not reporting the primary outcome of interest, two being meeting abstracts, and three for not focusing on ovarian cancer. Ultimately, 10 studies were included in the final review [16,17,18,19,20,21,22,23,24,25].

Table 2 summarized included studies that examined the NLR in differentiating between benign and malignant ovarian masses, including a total of 3675 patients across eight studies. Overall, the studies featured a diverse range of populations, with ages varying from less than 18 years to over 50 years. Cut-off values for NLR ranged from 1.17 to 3.35, with sensitivity values ranging from 42.65% to 91% and specificity values from 13% to 85.17%, indicating differing diagnostic performance. Notably, the majority of studies reported higher sensitivity values for detecting malignancy, although some had low specificity, suggesting a tendency to produce false positives in benign cases.

Multiple factors related to the patient and disease can alter the values for neutrophils and lymphocytes (and the NLR as well). In cases of ovarian masses, malignant tumors may raise neutrophil levels due to tumor-related inflammation, cytokine (inflammatory mediator) and immune system stimulation, which would raise the NLR. Simultaneously, levels of lymphocytes can fall due to cancer-related immune suppression, due to physiologic stress, or due to poor nutrition, which would raise the ratio even more. However, these are not exclusive to malignancy [16,17,18]. Age, menopausal status, associated comorbid inflammatory disease, infection (whether acute or chronic), and certain benign gynecological conditions, like endometrioma or a cyst of an ovary that is inflamed, can also modify the leukocyte profile, which could explain, at least in part, the reason that some studies described a phenomenon of large sensitivity but small specificity [16,17,19,20,23,24].

Furthermore, the variability of neutrophil and lymphocyte counts and the NLR cut-offs could be due to the methodological and clinical heterogeneity of the studies included in the review. The heterogeneity of the review could be due to differences in patient selection such as benign-only versus mixed benign/borderline groups, tumor subtype and stage (epithelial vs. non-epithelial), the timing of blood draws (preoperative inflammation, recent operative procedures, or illness), and the presence of post-operative conditions. Moreover, the neutrophil and lymphocyte counts could be altered due to the presence of cancer or as a result of non-cancer-related factors, such as the recent use of corticosteroids, chemotherapy, immunosuppression, or antibiotics, which could also explain the significant heterogeneity in this review. For these reasons, NLR should not be used in isolation to distinguish between malignant and benign ovarian tumors [16,17,18,19,20,21,22,23,24].

**Figure 2:**
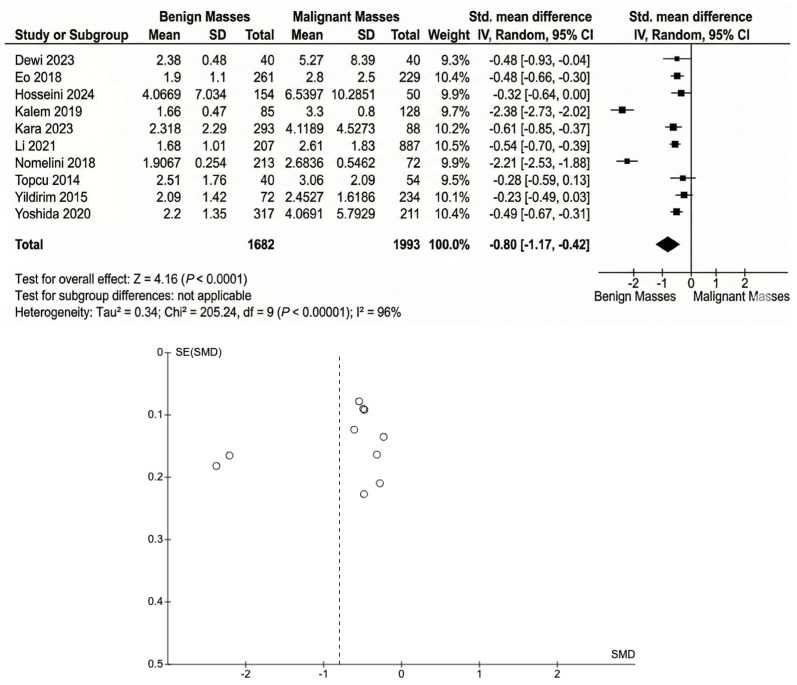
The pooled standard mean differences between benign and malignant ovarian masses. Reference: Dewi 2023 [16], Eo 2018 [17], Hosseini 2014 [20], Kalem 2019 [25], Kara 2023 [22], Li 2021 [24], Nomelini 2018 [19], Topcu 2014 [18], Yildirm 2015 [23] and Yoshida 2020 [21].

The pooled SMD for NLR (Figure 2) between benign and malignant ovarian masses was estimated to be −0.80 (95% CI: −1.17 to −0.42), indicating that malignant masses had a significantly higher mean than benign masses. The test for overall effect (Z = 4.16, *p* < 0.0001) showed a statistically significant difference between the two groups. There was considerable heterogeneity among the studies (I^2^ = 96%), suggesting substantial variability in the effect sizes across the included studies.

**Figure 3:**
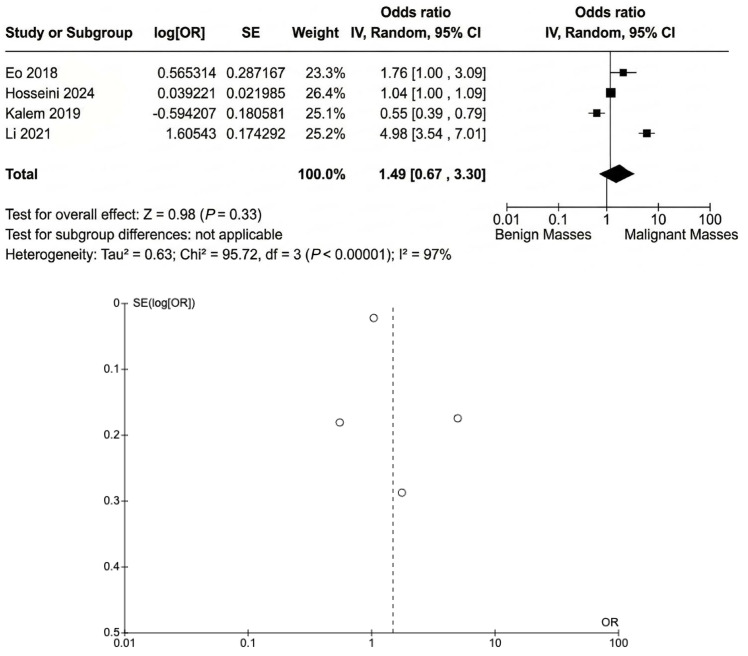
The odds of diagnosing malignant ovarian tumors based on the NLR. Reference: Eo 2018 [17], Hosseini 2024 [20], Kalem 2019 [25] and Li 2021 [24].

The pooled OR (Figure 3) for diagnosing malignant ovarian tumors based on the NLR was 1.49 (95% CI: 0.67 to 3.30). This suggested that a higher NLR may have been associated with an increased likelihood of diagnosing malignant ovarian tumors, although the result was not statistically significant (Z = 0.98, *p* = 0.33). There was substantial heterogeneity (I^2^ = 97%), indicating variation in the ORs across the studies.

**Figure 4:**
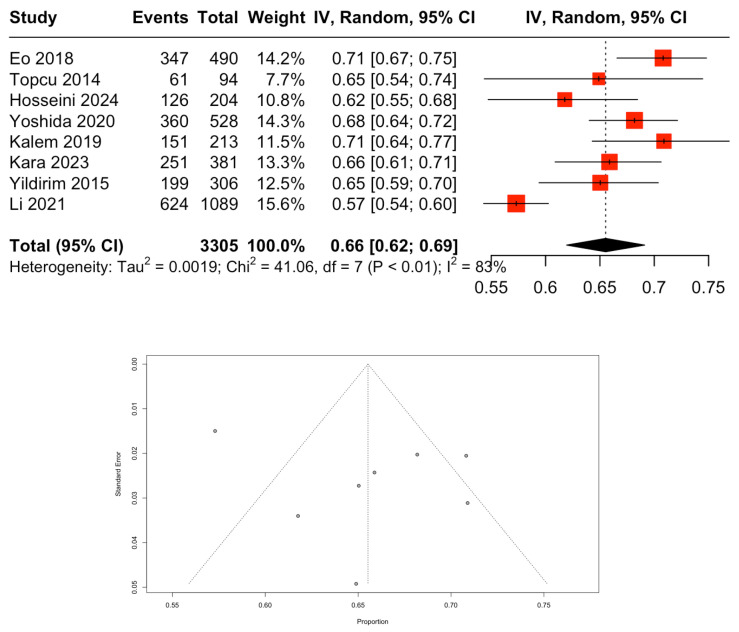
The pooled AUC for the NLR in distinguishing between benign and malignant ovarian tumors. Reference: Eo 2018 [17], Topcu 2014 [18], Hossenini 2024 [20], Yoshida 2020 [21], Kalem 2019 [25], Kara 2023 [22], Yildirim 2015 [23] and Li 2021 [24].

**Figure 5:**
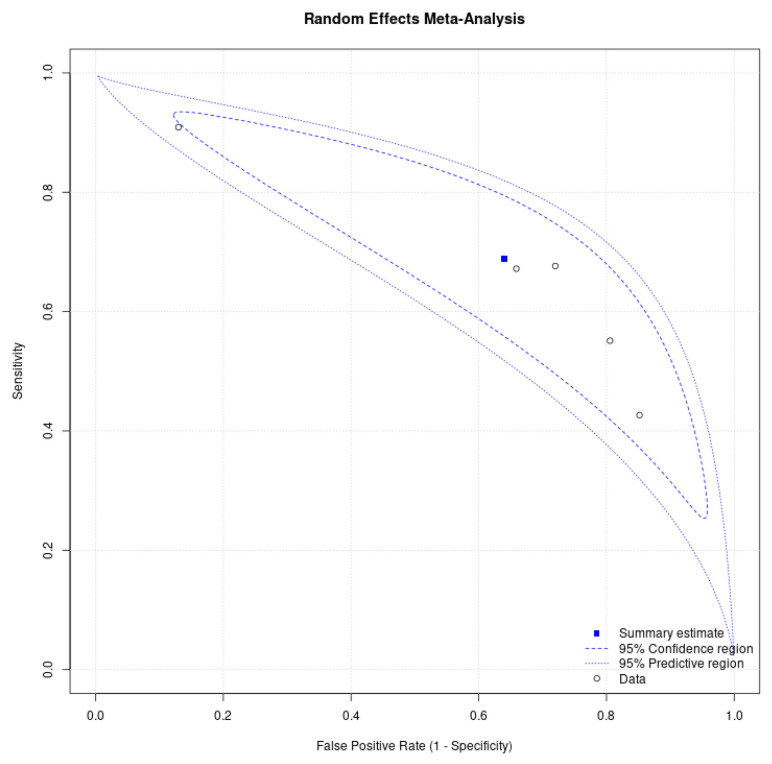
SROC for the NLR in distinguishing between benign and malignant ovarian tumors.

**Figure 6:**
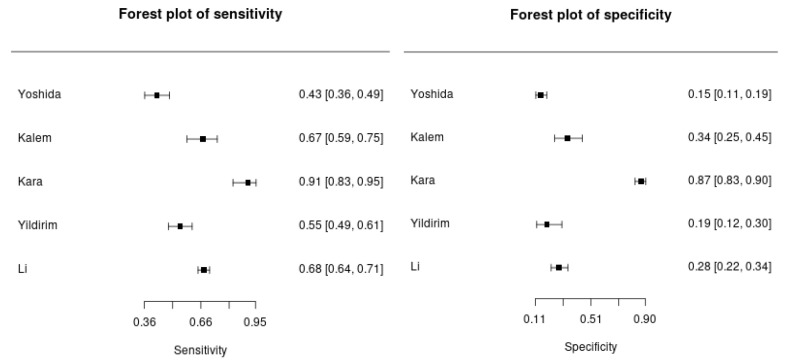
Sensitivity and specificity for the NLR in distinguishing between benign and malignant ovarian tumors.

The pooled analysis of the NLR for distinguishing between benign and malignant ovarian tumors showed moderate diagnostic performance, with an overall AUC of 0.66 (95% CI: 0.62–0.69). This indicated that NLR had some utility but was not highly accurate in this context (Figure 4). The high heterogeneity (I^2^ = 83%) across studies suggested significant variability in results, likely due to clinical or methodological differences. The SROC curve (Figure 5) further reflected moderate diagnostic ability, with sensitivity ranging from 0.43 to 0.91 and specificity from 0.15 to 0.87, indicating that while NLR was useful in detecting malignancy, it could produce false positives in benign cases (Figure 6).

**Figure 7:**
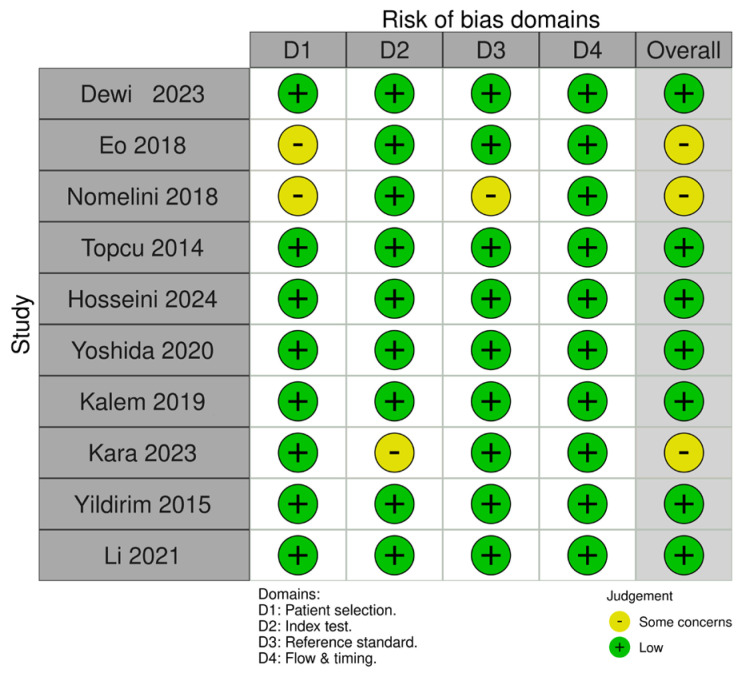
QUADAS-2 results for all included studies. Reference: Dewi 2023 [16], Eo 2018 [17], Nomelini 2018 [19], Topcu 2014 [18], Hossenini 2024 [20], Yoshida 2020 [21], Kalem 2019 [25], Kara 2023 [22], Yildirim 2015 [23] and Li 2021 [24].

The QUADAS-2 assessment in Figure 7 evaluated the risk of bias across four domains (patient selection, index test, reference standard, and flow and timing) in studies included for review. Most studies showed low risk of bias, with occasional concerns in the domains of patient selection (D1) and index test (D2). Overall, the majority of studies exhibit a favorable bias profile, with only a few marked as having “some concerns.”

## 4. Discussion

The findings of this systematic review and meta-analysis revealed a significant difference in NLR between benign and malignant ovarian masses, but not with an increased likelihood of malignancy. This elevation is attributed to the inflammatory response associated with tumor presence, where neutrophils increase and lymphocyte counts decrease [22]. The utility of NLR as a predictor for malignancy faces several limitations due to a lack of direct correlation with malignancy, group overlap, and issues with specificity. While studies have demonstrated differences in NLR between malignant and benign tumors, some show no significant variation between benign and borderline ovarian tumors, questioning its predictive accuracy [23,26]. The overlap between these groups undermines NLR as an independent marker. Additionally, although elevated NLR may suggest malignancy, its low specificity, such as 16% in one study, means many benign cases also exhibit high NLR due to inflammation, leading to false positives [22]. This is further complicated by the fact that elevated NLR can result from various non-cancerous inflammatory conditions, making it unreliable without considering other clinical factors [27].

Some of the findings should consider the biological aspects of systemic inflammation and the advancement of tumors. Malignant ovarian tumors create a strong and pro inflammatory response and microenvironment due to the production of various cytokines, chemokines, and other growth factors, which will initiate neutrophil production and recruiting, while lymphocyte mediated antitumor responses are suppressed [28]. Therefore, the elevated NLR resulting from an imbalance and host immune surveillance could denote inflammation [29]. This explains the NLR being more elevated in malignant than benign masses of the ovary [29]. It should be noted, however, that the inflammatory response is complex and varies with tumor burden, histological subtype and stage of the disease. For instance, more advanced malignancies are likely to elicit higher systemic inflammatory responses than early stage diseases, hence the inflammation may lead to even higher NLR which may explain the discrimination in some cohorts better than in other cohorts [30]. This is a partial explanation of the variability in the NLR performance in the studies that were considered.

When interpreting this review’s findings, it is essential to look at previous research on inflammation-based markers for ovarian tumors. In this meta-analysis, for NLR there was a significant difference concerning benign and malignant ovarian masses (pooled SMD = −0.80, 95% CI: −1.17 to −0.42). However, for the pooled odds ratio for malignancy, there was no statistical significance (OR = 1.49, 95% CI: 0.67–3.30). This shows the inconsistency between differences at the group level and a clinically significant prediction. Similar and previous studies have shown the NLR is usually elevated in malignant disease; however, the NLR shows a lack of discriminatory performance [31,32,33]. In the studies that are a part of this review, NLR cut-off values ranged widely between 1.17 and 3.35, and sensitivity and specificity values ranged respectively from 42.65% to 91% and from 13% to 85.17% [16]. This shows there is a large difference in diagnostic accuracy in different populations and settings. On the other hand, a threshold value of 3.35 gives a sensitivity of 55% and a specificity of 81%, which shows the same trade-off between sensitivity and specificity is observed, as expected [23].

The combined diagnostic measures for this review also coincide with previous studies indicating NLR as having moderate clinical usefulness for stand-alone usage as a marker [34]. In our analysis, the pooled AUC was 0.66 (95% CI: 0.62–0.69), with the SROC profile exhibiting the widest spans of sensitivity and specificity. In the previous literature, this has also been the case, with AUC values of roughly 0.65–0.70. This is consistent with NLR potentially assisting with risk stratification; however, when considered in isolation, it is not enough to make the critical call to order further evaluation for differentiated vs. malignant ovarian masses. This also shows that, while NLR is a valuable component in the preoperative evaluation, it offers limited discriminatory power as it is only available through a CBC [35]. Obscured NLR readings (secondary to, for example, a benign inflammatory condition that may artificially increase NLR and produce false-positive results) could overlook important clinical data that must be considered in conjunction with NLR. This is of the utmost importance when NLR is on record [36].

The pooled analysis of the AUC for the NLR demonstrated moderate discriminative power in distinguishing between benign and malignant ovarian masses, with an overall AUC of 0.66 (95% CI: 0.62–0.69). This result suggests that while NLR has some benefit as a diagnostic marker, it is not highly accurate, as indicated by the moderate AUC value. Despite its moderate AUC value, NLR remains valuable in clinical practice due to several factors. It is cost-effective, as it can be easily obtained from routine blood tests, and is highly accessible, allowing for quick measurement in both emergency and outpatient settings [37,38]. Additionally, NLR can complement other diagnostic tools by helping to stratify risk or guide further investigations when used alongside imaging studies and other biomarkers [39,40]. The SROC curve (0.43–0.91) further reinforces the moderate diagnostic ability of NLR. This suggests that although NLR may be effective at identifying malignant ovarian masses, it is less reliable at ruling out benign cases, leading to a higher likelihood of false positives. This limitation is particularly relevant when considering the clinical application of NLR as a diagnostic tool, as high sensitivity without corresponding specificity may result in unnecessary diagnostic interventions. Other studies have similarly found moderate diagnostic accuracy, with reported AUC values of around 0.65 to 0.70, reinforcing the notion that while NLR can be useful, it should be employed in conjunction with other diagnostic methods for improved accuracy in differentiating ovarian masses [20].

Inconsistencies with NLR performance in various studies may be due to the differences in participants, disease variety, and operational study techniques. Previous studies have captured varied population mixes with differing ratios of epithelial ovarian cancer, borderline tumors, endometrioma, and other benign adnexal masses that may affect the baseline level of inflammation [16,17,18,19,20,21,22,23,24]. Further, the study participants’ age and menopausal status varied widely and these factors may influence both the leukocyte count as well as the presumptive malignancy. There may also be heterogeneity due to operational differences such as a retrospective study design, varying infection/inflammatory comorbidity exclusions, and differences in the timing of blood draws. Despite these factors, there may be an association between increased NLR and ovarian malignancy [16,17,18,19,20,21,22,23,24]. There are not enough studies to have great confidence in this association but there are biologically encouraging data. Future studies should focus on the aforementioned issues. Additionally, NLR should be compared to other inflammatory markers and it should be determined if deficit models can be extended beyond the moderate accuracy demonstrated by NLR.

Most interesting of all is the non-significant pooled OR, despite the noticeable difference in mean NLR values between the groups. This indicates that although malignant masses may show, on average, larger NLR values, the magnitude and the consistency of the relation may be too weak to justify NLR as a potential independent predictor in a multitude of contexts [26]. A biomarker can present statistically significant differences across the groups, but at the same time, it can have very little discriminative value when it comes to the diagnosis of an individual patient [41]. This serves the clinician as an important distinction, since statistically significant differences at the level of the population do not mean that there will be a reliable clinical instrument to guide a decision. In this context, the moderate pooled AUC in this review is also in line with this and emphasizes the need to be cautious in the application of NLR cut-offs as a practice in this field [41].

The overlap between inflammatory responses due to malignancy and those due to benign gynecologic conditions warrants further consideration. Several benign ovarian conditions can cause increased neutrophil and altered lymphocyte counts [42]. Endometriosis/endometrioma, pelvic inflammation, and complicated cysts can also do this [42]. Because of this, NLR may become more nonspecific, leading to an increased possibility of a positive classification when being employed alone [30]. This is especially important in premenopausal patients, where benign inflammatory conditions of the ovaries are more common. In contrast in postmenopausal patients, they may have a higher baseline inflammatory pattern and higher pre-test probability of malignancy which may impact NLR diagnostic accuracy [43]. Thus, the patient’s age and menopausal status should be considered when NLR is interpreted and when designing studies to identify NLR cut-off values [30].

Study design variation may have also played a role in outcomes. Most studies included are retrospective which increases susceptibility to selection bias, incomplete clinical descriptions, and controls of confounding variables and clinical data; therefore, they are poorly constructed in controlling confounding variables, and clinical data may be inconsistent [44]. Variations in defining groups as benign and malignant, including borderline tumors, and variation in reference standards also impact diagnostic estimates. Some studies may have poor exclusion of participants with the infection of interest, hematologic disorders, or modifications to the leukocyte count within the timeframe of the study because of recent treatments [45]. These factors may obscure the actual relationship between NLR and malignancy of the ovaries.

While considering clinically, the data endorse NLR’s employment in the preoperative evaluations of adnexal masses as an adjunct, as opposed to an individual diagnostic test [46]. It is an inexpensive, quick, and routinely obtained value in a complete blood count, which, in resource-scarce settings, can provide a first-step risk stratification in the absence of advanced biomarkers, specialist imaging, and high-level clinical expertise [46]. With its moderate discriminative ability and variable specificity, the NLR must always be used in conjunction with the traditional diagnostic methods of ultrasonography, menopausal status, CA-125, and the relevant clinical prediction model [47]. It is posited that the NLR, used alongside other diagnostic modalities, will mitigate unnecessary surgical procedures due to false positive blood tests that reveal inflammation and, instead, provide a greater degree of diagnostic assurance [48].

There are many aspects that limit our study. There was substantial heterogeneity, which makes drawing definitive conclusions more difficult, and therefore limits generalization. Diagnostic heterogeneity and performance NLR are impacted by differences and cut-off values and demonstrate the need for established standards. The absence of study design uniformity which resulted in differences in inclusion criteria and patient populations particularly with biases, NLR values abstracted and pre vs. post operative NLR values are also biases and studies that are designed to be retrospective abstracted biases even more. Additionally, we were not able to perform a meta-regression analysis to control for potential confounding factors, which further limits the interpretation of between-study differences.

## 5. Conclusions

While NLR demonstrates promise in differentiating between benign and malignant ovarian tumors, its diagnostic performance is moderate, and considerable heterogeneity exists among studies. The variability in cut-off values, sensitivity, and specificity highlights the need for standardization in future research. Moreover, NLR should not be used in isolation but rather in conjunction with other diagnostic methods to improve accuracy and clinical decision-making.

## Figures and Tables

**Table 1:** Search protocol for this systematic review and meta-analysis.

Database	Search Terms Used
PubMed	“Neutrophil-to-lymphocyte”[All Fields] AND (“ratio”[All Fields] OR “ratio s”[All Fields] OR “ratioes”[All Fields] OR “ratios”[All Fields]) AND (“molecular weight”[MeSH Terms] OR (“molecular”[All Fields] AND “weight”[All Fields]) OR “molecular weight”[All Fields] OR “mass”[All Fields] OR (“cysts”[MeSH Terms] OR “cysts”[All Fields] OR “cyst”[All Fields] OR “neurofibroma”[MeSH Terms] OR “neurofibroma”[All Fields] OR “neurofibromas”[All Fields] OR “tumor s”[All Fields] OR “tumoral”[All Fields] OR “tumorous”[All Fields] OR “tumour”[All Fields] OR “neoplasms”[MeSH Terms] OR “neoplasms”[All Fields] OR “tumor”[All Fields] OR “tumour s”[All Fields] OR “tumoural”[All Fields] OR “tumourous”[All Fields] OR “tumours”[All Fields] OR “tumors”[All Fields])) AND (“ovary”[MeSH Terms] OR “ovary”[All Fields] OR “ovarium”[All Fields] OR (“ovarian”[All Fields] OR “ovarians”[All Fields]))
EMBASE	((benign ovarian mass or malignant ovarian mass) and Neutrophil-to-lymphocyte ratio).mp. [mp = title, abstract, heading word, drug trade name, original title, device manufacturer, drug manufacturer, device trade name, keyword heading word, floating subheading word, candidate term word]
SCOPUS	neutrophil-to-lymphocyte AND ratio AND mass OR tumor AND ovarium OR ovarian

**Table 2:** Demographic characteristics of the studies included in the review.

Study ID	Age, Years	Total Benign	Total Malignant	Details of Ovarian Benign Mass	Details of Ovarian Malignant Mass	NLR Cutoff	Diagnostic Details
Dewi 2023 [16]	<18 years: 1 vs. 0 18–49 years: 29 vs. 31 ≥50 years: 10 vs. 9	40	40	Ovarian cyst	Epithelial ovarian cancer	3.35	Sensitivity: 55% Specificity: 81%
Eo 2018 [17]	35 ± 21 vs. 54 ± 16	261	229	All benign mass	Epithelial ovarian cancer	2.64	Sensitivity: 56.8% Specificity: 77.4%
Nomelini 2018 [19]	Not specified	213	72	Ovarian cyst	Epithelial ovarian cancer	Not specified	Not specified
Topcu 2014 [18]	41.1 ± 7.85 vs. 53.5 ± 9.5	40	54	All benign mass	Epithelial ovarian cancer	Not specified	Not specified
Hosseini 2024 [20]	46.31 ± 13.21 vs. 54.26 ± 12.04	154	50	Benign ovarian cancer	Malignant ovarian cancer	2.61	Sensitivity: 60% Specificity: 63%
Yoshida 2020 [21]	Benign disease: 46.79 ± 16.17 Borderline ovarian tumor: 48.37 ± 16.22 Epithelial ovarian cancer: 59.22 ± 14.42 Non-epithelial ovarian cancer: 43.58 ± 18.17 Ovarian metastases: 53.45 ± 11.08	317	211	All benign mass	Borderline ovarian tumor Epithelial ovarian cancer Non-epithelial ovarian cancer Ovarian metastases	2.9	Sensitivity: 42.65% Specificity: 85.17%
Kalem 2019 [25]	29.14 ± 4.84 vs. 30.3 ± 4.41	85	128	Ovarian cyst	Endometrioma	1.73	Sensitivity: 67.2% Specificity: 65.9%
Kara 2023 [22]	Not specified	293	88	Benign ovarian cancer	Malignant ovarian cancer	1.17	Sensitivity: 91% Specificity: 13%
Yildirim 2015 [23]	Simple ovarian cyst median (IQR): 46 (25) Endometrioma median (IQR): 39 (14) Dermoid cyst median (IQR): 36.50 (22) Benign tumor median (IQR): 51 (20) Ovarian cancer median (IQR): 63.50 (23) Borderline tumor median (IQR): 61 (26)	72	234	Benign tumor	Epithelial ovarian cancer	3.35	Sensitivity: 55% Specificity: 81%
Li 2021 [24]	47.17 ± 14.66 vs. 54.23 ± 10.57	207	887	Benign ovarian masses	Malignant ovarian cancer	2.139	Sensitivity: 67.6% Specificity: 71.8%

## Data Availability

The data supporting the findings of this study are available from the corresponding author upon reasonable request.

## Abbreviations

The following abbreviations are used in this manuscript:

AUC	Area under the receiver operating characteristic curve
CA125	Cancer antigen 125
CI	Confidence interval
HE4	Human epididymis protein 4
I^2^	I-squared statistic (measure of heterogeneity)
NLR	Neutrophil-to-lymphocyte ratio
OR	Odds ratio
PRISMA	Preferred Reporting Items for Systematic Reviews and Meta-Analyses
QUADAS-2	Quality Assessment of Diagnostic Accuracy Studies-2
ROC	Receiver operating characteristic
SMD	Standard mean difference
SROC	Summary receiver operating characteristic
Z-test	Z test
